# P08 A multi-disciplinary approach to treating young patients with Complex Regional Pain Syndrome (CRPS)

**DOI:** 10.1093/rap/rkac067.008

**Published:** 2022-09-28

**Authors:** Robert Schneider, Glenda Dalton

**Affiliations:** Evelina London Children's Hospital, London, United Kingdom; Evelina London Children's Hospital, London, United Kingdom

## Abstract

**Introduction/Background:**

The Rheumatology service provides outpatient assessment and rehabilitative intervention for CYP with inflammatory/non-inflammatory pain conditions across South London and Southeast England.

CRPS remains poorly understood condition. Young people experience persistent severe and debilitating pain. The service receives approximately 20 referrals per annum, a figure increasing year-on-year.

Early recognition and intervention is key for successful outcomes, but referrals to specialist services may be delayed months from onset of symptoms.

No current national pathway to manage CRPS for paediatric patients.

The team are aiming to identify “gold standard” intervention for CYP with CRPS, using existing models. matching resource to need.

**Description/Method:**

Exact cause of CRPS unknown.

Injury sometimes trigger, but not 1/10 cases.

More common in women.

1.2/100,000 CYP (5-15 years old) in the UK; 15,000 new adult cases in adults pa (1 in 3,800).

Diagnosis mainly clinical. No specific test confirms CRPS. Mainly based on symptoms and physical examination.

Symptoms include:

usually single limb, can be widespread

pain (particularly allodynia), hypersensitivity, altered sensation

skin changes around affected area, e.g. sensitive to touch, change in temperature

swelling of limbs

hair & nail growth

functional impact

increased sweating

stiffness

increased anxiety, lower mood, depression

muscle weakness

Difficult to treat – no single treatment available

Duration of intervention varies from few weeks (mild cases) to indefinitely in some.

CRPS can impact on activities of daily living, mobility, school attendance, sleep, mood.

Early diagnosis and intervention is key.

Imperative need for MDT approach; enables CYP to start to live alongside pain and develop control over their lives.

CASE STUDY - 14F

Past Medical History = DDH and Perthes of L hip; last surgery October 2020 (metalwork removed)

2021 - Presented with significant pain, numbness and restricted movement in left leg. Admitted locally for 6-day investigation. Labelled “very complex”. No improvement upon discharge.

Limited mobility – using wheelchair

Reduced school attendance (30%)

Not wearing shoe/sock (left foot)

Limited socialisation

Affecting mood and mental well-being

2022 Referral to RhEve

Attended ELCH One-Stop Clinic; met Rheumatologist

Reassurance, understanding and validation + robust diagnosis of CRPS

Referred to Physio/OT for intervention

Seen within 1/52 by Physio/OT

Repeated CRPS messages – ensured understanding; reassurance that team “know” how to treat; not complex for us!

Focus not on “fixing” pain

Use of breathing/distraction techniques; encouraged weight-bearing, movement, desensitisation and mobility

**Discussion/Results:**

CASE STUDY INTERVENTION OUTCOMES [after one x 2 hour therapy session]:

Touching own leg

Noticed colour change in L leg/foot

Actively started to move toes

Took partial weight through L heel using crutches

Negotiated stairs

Reported to feel “confident” enough to be able to work on strategies at home

Trialled wearing a soft shoe on her L foot

Learnt breathing techniques and was able to use distraction

Able to set some functional goals, e.g. school attendance, playing football, seeing friends

From our extensive experience, we understand that:

Intervention begins with the individual) - “Being healthy is more than just not being ill - it's about our physical, mental and emotional wellbeing”

MDT approach is the most successful treatment for CRPS.

This patient group demands a great deal of resource (e.g. clinics, inpatient admissions, therapy intervention time, liaison between ELCH and local teams)

A confident, robust diagnosis is essential to support the young person and family in engaging with treatment intervention, moving from pre-contemplation to contemplation stage of change

The CYP & their family must understand the diagnosis and show readiness for rehabilitation in order to move to the preparation stage of change

Therapeutic intervention is most likely to be successful when the patient has engaged with treatment and has started to gain control of their symptoms, thereby moving from preparation towards action

Our hypothesis is that by being aware of the stages of change and delivering interventions at the “correct” time the team can facilitate improved and quicker outcomes with the patient’s rehabilitation.

**Key learning points/Conclusion:**

The young people that we see:

Need confident & robust diagnoses delivered with empathy

Benefit from time and space to process complex diagnoses

Gain from continual opportunities for learning and asking questions

Deserve to feel listened to, and receive reassurance and validation with empathy and compassion

The need for a tailored approach for each individual CYP as each individual’s needs differ! Clinicians need to be flexible, e.g. duration and timings of appointments

Value multi-disciplinary working – this is also an opportunity for clinicians to learn and feel supported

We have a responsibility to provide learning opportunities and mentoring/support to local network colleagues

WHAT NEXT? To potentially develop National guidelines for Child and Young Person (CYP) with other stake holders.

